# Drinking or Smoking While Breastfeeding and Later Academic Outcomes in Children

**DOI:** 10.3390/nu12030829

**Published:** 2020-03-20

**Authors:** Louisa Gibson, Melanie Porter

**Affiliations:** Faculty of Medicine, Health and Human Sciences, Department of Psychology, Macquarie University, Balaclava Road, North Ryde, NSW 2109, Australia; melanie.porter@mq.edu.au

**Keywords:** breastfeeding, alcohol, cigarettes, smoking, drinking, academic achievement, numeracy, literacy

## Abstract

Alcohol consumed by breastfeeding mothers has been associated with reduced grammatical comprehension and cognition in children. This study examined whether drinking or smoking while breastfeeding was associated with reductions in Australian National Assessment Program–Literacy and Numeracy assessments. Data was sourced from The Growing Up in Australia Study. This is an ongoing longitudinal study of 5107 infants and mothers recruited in 2004 and followed over time every two years. Multivariable linear regression found that maternal alcohol consumption at study entry was associated with reductions in Grade 3 (age 7–10 years) National Assessment Program–Literacy and Numeracy writing (b = −1.56, 95% CI: −2.52; −0.60, *p* = 0.01), spelling (b = −2.06, 95% CI: −3.31; −0.81, *p* < 0.0001) and grammar and punctuation (b = −2.11, 95% CI: −3.59; −0.64, *p* = 0.01) scores, as well as Grade 5 (age 9–11 years) spelling scores (b = −1.58, 95% CI: −2.74; −0.43, *p* = 0.03) in children who had been breastfed at any time. This was not evident in babies who had never breastfed, or in the smaller group of infants who were actively breastfeeding at study entry. Smoking was not associated with any outcome variable. Drinking alcohol while breastfeeding may result in dose-dependent reductions in children’s academic abilities. While reductions are small, they may be of clinical significance if mothers drink large quantities. Further analyses are planned to assess developmental, physical and behavioural outcomes in children.

## 1. Introduction

Prenatal alcohol and tobacco have been associated with poorer academic achievement in children following birth [1,2,3,4]. While alcohol [5] and nicotine [6] can transfer to infants through breastmilk, the cognitive impacts have only recently been explored [7], and the academic effects on children are largely unknown.

Although the World Health Organization (WHO) recommends avoiding drugs and alcohol while breastfeeding [8], not all health bodies advise complete abstinence [9]. Many women report consuming alcohol (12–83%) [10,11,12,13,14,15,16,17] and smoking tobacco (7–16%) [14,18] while breastfeeding, and actual numbers may be higher due to underreporting [19]. Maternal age, education, income and breastfeeding duration are the most frequent factors associated with use [14,20].

Nicotine transfers to breastmilk [6], where it can reduce milk production and change composition and taste [21]. While no cognitive reductions in relation to tobacco use during lactation have been observed [7], lower birth weight [22] and earlier weaning [23] have been associated with both prenatal tobacco use and reduced cognitive abilities [24,25]. Observed dose-dependent reductions in milk iodine content [26] are also a potential mechanism by which nicotine use during lactation could theoretically impair cognitive and academic achievement [27].

Alcohol also transfers to breastmilk [28] where it reduces milk production [29] and alters sleeping and feeding patterns [30]. In one case study, the pseudo-Cushing symptoms of an infant abated once the high maternal alcohol consumption during lactation was ceased [31]. Additionally, several rat studies have found an association between maternal alcohol consumption during lactation and both physiological and cognitive aspects of brain development in offspring [32,33,34,35,36].

Findings from human studies of alcohol consumption while breastfeeding and either cognitive or academic outcomes are mixed. While Little et al. [37] found reduced psychomotor scores at one year in babies whose mothers drank while breastfeeding, more recent studies found no reduction in developmental scores [17,38]. In contrast to the poorer grammatical comprehension of children whose mothers consumed alcohol during lactation [39], Gibson and Porter [7] found no dose-dependent relationship between maternal alcohol use while breastfeeding and either vocabulary or early literacy screening scores.

A dose-dependent reduction in non-verbal abstract reasoning ability has been observed in children of mothers who drank alcohol while breastfeeding. This relationship was independent of pregnancy alcohol, as well as many socio-economic and known cognition-impacting factors. No association was observed in babies who had never breastfed, suggesting that reductions may be due to a direct relationship between alcohol and breastmilk, rather than environmental or social aspects surrounding alcohol consumption [7]. This supports the suggestion that alcohol consumed while breastfeeding may impact children’s academic achievement.

The aim of the current study was to assess whether drinking alcohol or smoking cigarettes during lactation adversely impacts academic outcomes in children. It was hypothesised that alcohol and tobacco use would result in lower National Assessment Program–Literacy and Numeracy (NAPLAN) scores in a dose dependent manner that was independent of pregnancy use.

## 2. Materials and Methods 

### 2.1. Ethics

Ethics approval was obtained from Macquarie University Human Research Ethics Committee (approval code 5201822862659, 7 April 2017).

### 2.2. Study Design, Data Source and Study Cohort

Details of the study have been described previously [7]. Briefly, data was sourced from an ongoing longitudinal study titled Growing Up in Australia: The Longitudinal Study of Australian Children (LSAC) [40]. The study cohort available to the authors for analysis comprised 5107 infants and caregivers from LSAC born in 2003–2004 and recruited in 2004. Participants were followed over time every two years in data “waves”. Wave 1 reflects study entry. Demographic, lifestyle, cognitive, academic and developmental variables were collected at each of the six waves available to authors for analysis [41,42]. Data was also linked to NAPLAN [43]. Further recruitment details are available in LSAC Technical Paper No1 [44].

### 2.3. Breastfeeding

Caregivers were asked whether infants were being breastfed at Wave 1 and whether they had ever been breastfed [45]. This allowed the sample to be divided into Wave 1 (study entry) breastfeeding babies and babies who had been breastfed at any time (babies who were breastfeeding at Wave 1 combined with babies who had been breastfed previously but had stopped by study entry) for analysis. A separate group of babies who had never been breastfed was also analysed. These groups were used to replicate previous findings [7].

### 2.4. Predictor Variables

Mothers were asked a modified version of the alcohol use disorders identification test (AUDIT) Alcohol Consumption Questions (AUDIT-C) [46,47] at Wave 1 (study entry). Pregnancy alcohol was recorded retrospectively as the number of days per week mothers drank alcohol and the quantity they consumed on each occasion. Rather than averaging alcohol consumption days across trimesters as previously described [7], data was separated into trimesters to account for possible trimester effects. Mothers were also asked how many cigarettes they smoked on average per day at Wave 1 and during pregnancy. Further details, including a full description of the modified AUDIT-C, have been reported previously [7]. Smoking or drinking at later waves was not included in any analyses, since use of alcohol or tobacco at later waves does not reflect maternal use during lactation.

### 2.5. Outcome Variables

The NAPLAN assessments are a government academic monitoring program given to all Australian children in academic grades 3, 5, 7 and 9. It comprises annual tests of reading, writing, language conventions (spelling, grammar and punctuation) and numeracy. Results of NAPLAN are scaled, and are used to evaluate pedagogy and provide information on the individual performance of children [48].

The available NAPLAN scaled scores [49] were as follows (higher scores indicate better performance):

- Grades 3 and 5 reading scores
- Grades 3 and 5 writing scores
- Grades 3 and 5 spelling scores
- Grades 3 and 5 grammar and punctuation scores
- Grades 3 and 5 numeracy scores

### 2.6. Control Variables

Details regarding control variables have been described previously [7]. Briefly, they included sex (referent category: male), child age (at the time of NAPLAN assessment), maternal age (Wave 1), combined family income (Wave 1), maternal education (Wave 1), birthweight, and breastfeeding duration, since these have all been associated with cognitive or academic outcomes in children [24,25,50,51,52,53]. Learning difficulties and head injuries were omitted, since they were recorded at times not directly corresponding to outcome variables. In analyses of babies who had been breastfed at any time, breastfeeding status at Wave 1 (current or prior) was added as a control variable (reference category: current), since modified AUDIT-C scores were not contemporaneous to lactation in the group of babies who had ceased breastfeeding at the time of study entry. Each variable was only included from measurement at one data wave to prevent issues with multicollinearity.

Despite NAPLAN tests being English-reliant, it has been repeatedly observed that children from language backgrounds other than English (LBOTE) perform better than children from English speaking backgrounds in NAPLAN writing, spelling, grammar and punctuation, and numeracy tests [54]. Since it has been suggested that this may be an artefact of bias within the usual LBOTE measures [55], LBOTE was omitted as a control variable.

### 2.7. Statistical Analyses

Data was analysed using IBM SPSS version 24. Missing data from all included variables was imputed using multiple imputation (MI). Full details of the MI method have been described previously [7]. Changes to this method included using 35 imputations, since the highest proportion of missing data for any variable was 35% (Table 1 and Table 2 and prior [7]). When missing data is <50%, matching the imputation number to missing data percentage increases efficiency and replicability of data [56].

Multivariable linear regression analyses were performed including each of the predictor and control variables separately for each outcome variable. Although NAPLAN data was skewed (Kolmogorov-Smirnov D = 0.30–0.10, *p* < 0.001), linear regression has been shown to be robust to data skew in large sample sizes, and a valid method of statistical analysis [57,58,59]. The regression method used was identical to that described previously [7], including the use of the Benjamini-Hochberg procedure [60] to correct for Type I error (α = 0.05, 2-tailed).

## 3. Results

### 3.1. Descriptive Statistics (Prior to MI)

Additional descriptive statistics not previously reported [7] are shown in Table 1 and Table 2.

### 3.2. Power Analyses

Only data from biological mothers and their children were included. Following MI (d = 0.2, α = 0.05), a sample size of 2008 babies who were actively breastfeeding at Wave 1 provided >99% power with 14 independent variables. With 4679 babies who had been breastfed at any time, >99% power was achieved with 15 independent variables. Only one predictor variable (modified AUDIT-C scores) was utilised in the group of babies who had never been breastfed (n = 411) to maximise power at 80% [61].

### 3.3. Wave 1 Maternal Alcohol Consumption and Tobacco Smoking Prior to MI

In addition to previously described statistics [7], Wave 1 modified AUDIT-C scores and cigarettes smoked per day were analysed separately between babies who were breastfeeding at Wave 1 (study entry) and babies who had previously breastfed (prior to study entry). Results are available in Supplementary materials S1.

### 3.4. Pattern of Missing Data Prior to MI

Little’s Missing Completely at Random (MCAR) test found that data was not MCAR: χ^2^ =7322.89, df = 4922, *p* ≤ 0.0001. Previous LSAC data analysis found that more poorly educated parents tended to drop out of the study [62]. This suggests that data was missing at random and suitable for MI [63].

### 3.5. NAPLAN Reading Scores

Additional results available in Supplementary materials S2.

#### 3.5.1. Babies Breastfeeding at Wave 1

For grade 3 scores, the model explained 10–13% of variance across imputations. Analysis conducted on the original sample prior to MI explained 10% of variance. Older child age, female sex, increased maternal education and higher family income were associated with higher scores.

For grade 5 scores, the model explained 10–13% of variance across imputations. Analysis conducted on the original sample prior to MI explained 11% of variance. Increased maternal education and higher family income were associated with higher scores. No other statistically significant associations were observed.

#### 3.5.2. Babies Who Had Been Breastfed at Any Time

For grade 3 scores, the model explained 11–13% of variance across imputations. Analysis conducted on the original sample prior to MI explained 13% of variance. Older child age, older maternal age, increased maternal education, higher family income, female sex and prior breastfeeding status were associated with higher scores. 

For grade 5 scores, the model explained 11–13% of variance across imputations. Analysis conducted on the original sample prior to MI explained 11% of variance. Older child age, older maternal age, increased maternal education, higher family income, female sex and prior breastfeeding status were associated with higher scores. No other statistically significant results were observed.

### 3.6. NAPLAN Writing Scores

Additional results available in Supplementary materials S3.

#### 3.6.1. Babies Breastfeeding at Wave 1

For grade 3 scores, the model explained 12–15% of variance across imputations. Analysis conducted on the original sample prior to MI explained 14% of variance. Older child age, increased maternal education, higher family income and female sex were associated with higher scores.

For grade 5 scores, the model explained 12–14% of variance across imputations. Analysis conducted on the original sample prior to MI explained 12% of variance. Increased maternal education, higher family income and female sex were associated with higher scores. No other statistically significant results were observed.

#### 3.6.2. Babies Who Had Been Breastfed at Any Time

For grade 3 scores, the model explained 14–16% of variance across imputations. Analysis conducted on the original sample prior to MI explained 14% of variance. Older child age, increased maternal education, higher family income and female sex were associated with higher scores. Higher modified AUDIT-C scores and increased number of cigarettes smoked during pregnancy were associated with lower scores (Table 3).

For grade 5 scores, the model explained 12–15% of variance across imputations. Analysis conducted on the original sample prior to MI explained 13% of variance. Older maternal age, increased maternal education, higher family income and female sex were associated with higher scores. Increased number of alcoholic drinks consumed during pregnancy was associated with lower scores. No other statistically significant results were observed.

#### 3.6.3. Babies Who Had Never Been Breastfed

For grade 3 scores, the model accounted for 2–15% variance across imputations. Analysis conducted on the original sample prior to MI explained 2% of variance. Modified AUDIT-C scores were not associated with writing scores (b = −1.79, 95% CI: −4.40; 0.82, *p* = 0.18).

### 3.7. NAPLAN Spelling Scores

Additional results available in Supplementary materials S4.

#### 3.7.1. Babies Breastfeeding at Wave 1

For grade 3 scores, the model explained 8–10% of variance across imputations. Analysis conducted on the original sample prior to MI explained 9% of variance. Older child age, increased maternal education, higher family income and female sex were associated with higher scores.

For grade 5 scores, the model explained 7–9% of variance across imputations. Analysis conducted on the original sample prior to MI explained 7% of variance. Increased maternal education, higher family income and female sex were associated with higher scores. No other statistically significant results were observed.

#### 3.7.2. Babies Who Had Been Breastfed at Any Time

For grade 3 scores, the model explained 9–11% of variance across imputations. Analysis conducted on the original sample prior to MI explained 11% of variance. Older child age, increased maternal education, higher family income and female sex were associated with higher scores. Higher modified AUDIT-C scores and increased number of cigarettes smoked during pregnancy were associated with lower scores (Table 4).

For grade 5 scores, the model explained 7–10% of variance across imputations. Analysis conducted on the original sample prior to MI explained 9% of variance. Increased maternal education, higher family income and female sex were associated with higher scores. Higher modified AUDIT-C scores and increased number of cigarettes smoked per day while pregnant were associated with lower scores. No other statistically significant results were observed (Table 5).

#### 3.7.3. Babies Who Had Never Been Breastfed

For grade 3 scores, the model accounted for 4–13% of variance across imputations. Analysis conducted on the original sample prior to MI explained 0.7% of variance. Modified AUDIT-C scores were not associated with spelling scores (b = −2.05, 95% CI: −5.10; 0.99, *p* = 0.19).

For grade 5 scores, the model accounted for 3–17% variance across imputations. Analysis conducted on the original sample prior to MI explained 3% of variance. Modified AUDIT-C scores were not associated with spelling scores (b = −1.57, 95% CI: −4.47; 1.32, *p* = 0.29).

### 3.8. NAPLAN Grammar and Punctuation Scores

Additional results available in Supplementary materials S5.

#### 3.8.1. Babies Breastfeeding at Wave 1

For grade 3 scores, the model explained 10–13% of variance across imputations. Analysis conducted on the original sample prior to MI explained 11% of variance. Older child age, increased maternal education, higher family income and female sex were associated with higher scores.

For grade 5 scores, the model explained 11–15% of variance across imputations. Analysis conducted on the original sample prior to MI explained 13% of variance. Increased maternal education, higher family income and female sex were associated with higher scores. No other statistically significant scores were observed.

#### 3.8.2. Babies Who Had Been Breastfed at Any Time

For grade 3 scores, the model explained 12–13% of variance across imputations. Analysis conducted on the original sample prior to MI explained 13% of variance. Older child age, older maternal age, increased maternal education, higher family income and female sex were associated with higher scores. Higher modified AUDIT-C scores and increased number of cigarettes smoked during pregnancy were associated with lower scores (Table 6).

For grade 5 scores, the model explained 12–14% of variance across imputations. Analysis conducted on the original sample prior to MI explained 13% of variance. Older maternal age, increased maternal education, higher family income and female sex were associated with higher scores. Increased number of cigarettes smoked during pregnancy were associated with lower scores. No other statistically significant results were observed.

#### 3.8.3. Babies Who Had Never Been Breastfed

For grade 3 scores, the model accounted for 0.1–12% of variance across imputations. Analysis conducted on the original sample prior to MI explained 0% of variance. Modified AUDIT-C scores were not associated with grammar and punctuation scores (b = −1.04, 95% CI: −4.79; 2.71, *p* = 0.59).

### 3.9. NAPLAN Numeracy Scores

Additional results available in Supplementary materials S6.

#### 3.9.1. Babies Who Were Breastfeeding at Wave 1

For grade 3 scores, the model explained 11–14% of variance across imputations. Analysis conducted on the original sample prior to MI explained 13% of variance. Older child and maternal age, increased maternal education and higher family income were associated with higher scores.

For grade 5 scores, the model explained 11–14% of variance across imputations. Analysis conducted on the original sample prior to MI explained 13% of variance. Increased maternal education, higher family income and male sex were associated with higher scores. No other statistically significant results were observed.

#### 3.9.2. Babies Who Had Breastfed at Any Time

For grade 3 scores, the model explained 11–14% of variance across imputations. Analysis conducted on the original sample prior to MI explained 13% of variance. Older child age, older maternal age, increased maternal education, higher family income, higher birth weight and male sex were associated with higher scores. 

For grade 5 scores, the model explained 11–14% of variance across imputations. Analysis conducted on the original sample prior to MI explained 13% of variance. Older maternal age, increased maternal education, higher family income, higher birth weight and male sex were associated with lower scores. No other statistically significant results were observed.

## 4. Discussion

A dose-dependent association between increased or riskier maternal alcohol consumption while breastfeeding and decreased academic scores in children was observed in both grades 3 and 5. Greater or riskier maternal alcohol intake was associated with decreased grade 3 writing, spelling, and grammar and punctuation scores. These findings were maintained for grade 5 spelling scores, and were independent of prenatal alcohol consumption, sex, child age, maternal age, income, birth weight, breastfeeding duration, breastfeeding status. and pregnancy and breastfeeding tobacco smoking. 

These findings support and extend upon the findings of Gibson and Porter [7], who found dose-dependent reductions in children’s abstract reasoning abilities at age 6–7 years. They are also consistent with the poorer grammatical comprehension observed in children of mothers who consumed alcohol while breastfeeding [39]. Furthermore, child [53] and maternal age [50], income [52], maternal education [52], birthweight [24], sex [51], tobacco smoking [1,4] and alcohol consumption during pregnancy [2,3] were all associated with academic scores in manners consistent with prior research. This supports the validity of the statistical models.

No relationship between maternal alcohol consumption and any of the academic scores was found in babies who had never breastfed. This suggests that exposure to alcohol through breastfeeding was responsible for observed reductions, and not psychosocial or environmental factors surrounding maternal alcohol consumption. Alternatively, although power was maximised, it is possible that the sample size of babies who had never breastfed was too small to detect an association. 

There was no relationship between maternal alcohol consumption and any outcome variable in babies actively breastfeeding at study entry. As suggested by Gibson and Porter [7], this may be related to the small sample size. There was no observed association between maternal tobacco smoking and any of the academic outcomes in any of the breastfeeding groups or ages.

Unlike Gibson and Porter [7], who observed cognitive reductions in children aged 6–7, but not 10–11 years, the current study found reductions at both earlier (grade 3: 7–10 years) and later (grade 5: 9–11 years) ages. While the association in grade 5 children was only evident in spelling scores, it suggests that the effects of maternal alcohol consumption during lactation on children’s academic achievement may persist as the child ages.

Although these findings support the suggestion that drinking alcohol while breastfeeding can reduce academic outcomes in children, the relationship was small and may have little clinical significance unless mothers consume large quantities of alcohol or regularly binge drink. Possible neurotoxic effects [32,33,34] in the child are only one possible mechanism of action. Alcohol consumed during lactation can also reduce milk production [29] and change the feeding and sleeping patterns of the infant [30]. It is therefore possible that early nutritional deficits or reduced exposure to environmental stimuli could be responsible.

There are several limitations to the study. The measurement of alcohol consumption during pregnancy and breastfeeding differed, making direct comparisons difficult. The pregnancy measure was also retrospective and did not include a measure of binge drinking. While breastfeeding alcohol was measured using a score based on a scale developed by the WHO [46], it did not take the timing of alcohol consumption relative to infants’ milk consumption into account. As such, it was not possible to calculate the exact alcohol intake of infants. Furthermore, in the group of children who had ceased breastfeeding by the time of study entry, the mother’s modified AUDIT-C score was not contemporaneous to their breastfeeding. Although this was controlled for in analyses, it is possible that it still confounded findings. Future research should seek to comprehensively and contemporaneously measure alcohol intake relative to breastfeeding.

## 5. Conclusions

Increased or riskier maternal alcohol consumption during lactation was associated with dose-dependent reductions in grade 3 (age 7–10 years) writing, spelling, and grammar and punctuation scores, as well as grade 5 (age 9–11 years) spelling scores. No association was observed in babies who had never been breastfed. These findings were consistent with the poorer grammatical comprehension [39] and dose-dependent reduction in abstract reasoning ability observed in children of mother’s who drank alcohol while breastfeeding [7]. This suggests that alcohol consumed during lactation can impact the academic achievement of children. Future research should seek to clarify this relationship by quantifying the amount of alcohol ingested by infants via breastmilk relative to later academic outcomes.

## Figures and Tables

**Table 1:** Descriptive statistics for wave 1 breastfeeding status prior to multiple imputation (MI).

Breastfeeding Status at Wave 1	*N* (%)
Breastfeeding at Wave 1	2007 (39.3%)
Previously breastfed prior to Wave 1	2676 (52.4%)
Never breastfed	420 (8.2%)
Missing	4 (0.1%)

**Table 2:** Descriptive statistics for child’s age and NAPAN scores prior to MI.

Variable	*N*	Mean (SD)	Median	Range	Interquartile Range	Missing Data *N* (%)
Child’s age Grade 3 (months)	3790	102.48 (4.40)	103.00	82.00–121.00	6.00	1317 (25.8)
Child’s age Grade 5 (months)	3474	126.17 (3.93)	126.00	112.00–134.00	6.00	1633 (32.0)
NAPLAN Grade 3 Reading scores	3651	434.13 (91.46)	430.90	97.70–735.60	124.20	1456 (28.5)
NAPLAN Grade 3 Writing scores	3649	421.54 (64.70)	428.30	0.00–640.10	77.50	1458 (28.5)
NAPLAN Grade 3 Spelling scores	3655	416.30 (79.53)	418.20	178.70–617.00	103.90	1452 (28.4)
NAPLAN Grade 3 Grammar and punctuation scores	3655	435.47 (95.56)	431.10	0.00–729.00	121.80	1452 (28.4)
NAPLAN Grade 3 Numeracy scores	3642	407.98 (74.63)	410.20	170.60–739.00	102.00	1465 (28.7)
NAPLAN Grade 5 Reading scores	3352	517.45 (79.39)	516.80	86.90–811.40	98.50	1755 (34.4)
NAPLAN Grade 5 Writing scores	3337	477.87 (66.09)	477.40	94.50–702.00	71.00	1770 (34.7)
NAPLAN Grade 5 Spelling scores	3354	502.14 (72.88)	505.20	283.80–697.40	96.90	1753 (34.3)
NAPLAN Grade 5 Grammar and punctuation scores	3354	517.56 (84.36)	513.70	81.50–827.80	104.90	1753 (34.3)
NAPLAN Grade 5 Numeracy scores	3336	501.26 (70.99)	499.30	122.00–836.90	86.80	1771 (34.7)

**Table 3:** Grade 3 NAPLAN writing scores (babies who had been breastfed at any time).

Variable	B	SE	95% CI	*P* Value	Benjamini-Hochberg *P* Value
Child’s sex	28.17	2.06	24.12–32.22	<0.0001	<0.0001
Combined family income *	−3.86	0.47	−4.78–(−)2.93	<0.0001	<0.0001
Mother’s level of education	6.00	0.72	4.58–7.42	<0.0001	<0.0001
Child’s age (months)	1.13	0.24	0.66–1.60	<0.0001	<0.0001
Mother’s modified AUDIT-C score Wave 1	−1.56	0.49	−2.52–(−)0.60	<0.0001	0.01
Average daily cigarettes while pregnant	−1.63	0.51	−2.64–(−)0.63	<0.0001	0.01
Mother’s age Wave 1	0.42	0.21	0.02–0.82	0.04	0.09
Pregnancy: 2nd trimester days per week drank alcohol	5.48	3.50	−1.40–12.36	0.12	0.22
Child’s birth weight (grams)	<0.0001	<0.0001	<0.0001–0.01	0.16	0.26
Breastfeeding duration (days)	0.01	0.01	<0.0001–0.02	0.19	0.29
Pregnancy: 1st trimester days per week drank alcohol	−2.35	2.38	−7.04–2.34	0.33	0.44
Pregnancy: Average number of drinks	1.25	2.24	−3.16–5.65	0.58	0.68
Breastfeeding status (currently or previously breastfed)	−1.15	2.15	−5.37–3.08	0.59	0.68
Pregnancy: 3rd trimester days per week drank alcohol	−1.38	2.98	−7.23–4.47	0.64	0.69
Mother’s Average daily cigarettes Wave 1	0.11	0.33	−0.55–0.76	0.75	0.75
Intercept	236.51	27.32	182.79–290.23	<0.0001	N/A

* Higher scores indicate lower income.

**Table 4:** Grade 3 NAPLAN spelling scores (babies who had been breastfed at any time).

Variable	B	SE	95% CI	*P* Value	Benjamini-Hochberg *P* Value
Child’s sex	23.07	2.46	18.25–27.89	<0.0001	<0.0001
Combined family income *	−3.39	0.57	−4.51–(−)2.28	<0.0001	<0.0001
Mother’s level of education	7.01	0.92	5.21–8.81	<0.0001	<0.0001
Child’s age (months)	1.49	0.28	0.94–2.03	<0.0001	<0.0001
Mother’s modified AUDIT-C score Wave 1	−2.06	0.63	−3.31–(−)0.81	<0.0001	<0.0001
Average daily cigarettes while pregnant	−1.60	0.56	−2.71–(−)0.50	0.01	0.01
Mother’s age Wave 1	0.50	0.25	0.01–0.99	0.05	0.10
Pregnancy: 2nd trimester days per week drank alcohol	3.24	4.57	−5.74–12.23	0.48	0.87
Pregnancy: 3rd trimester days per week drank alcohol	2.07	3.74	−5.28–9.42	0.58	0.87
Breastfeeding duration (days)	<0.0001	0.01	−0.01–0.02	0.58	0.87
Pregnancy: Average number of drinks	−1.07	2.74	−6.46–4.32	0.70	0.88
Pregnancy: 1st trimester days per week drank alcohol	−1.06	2.95	−6.86–4.73	0.72	0.88
Child’s birth weight (grams)	<0.0001	<0.0001	<0.0001–0.01	0.76	0.88
Mother’s Average daily cigarettes Wave 1	0.03	0.43	−0.83–0.88	0.95	0.99
Breastfeeding status (currently or previously breastfed)	0.04	2.61	−5.08–5.16	0.99	0.99
Intercept	201.31	31.58	139.36–263.26	<0.0001	N/A

* Higher scores indicate lower income.

**Table 5:** Grade 5 NAPLAN spelling scores (babies who had been breastfed at any time).

Variable	B	SE	95% CI	*P* Value	Benjamini-Hochberg *P* Value
Child’s sex	18.82	2.37	14.18–23.47	<0.0001	<0.0001
Combined family income *	−2.96	0.51	−3.97–(−)1.95	<0.0001	<0.0001
Mother’s level of education	6.89	0.86	5.21–8.57	<0.0001	<0.0001
Mother’s modified AUDIT-C score Wave 1	−1.58	0.59	−2.74–(−)0.43	0.01	0.03
Average daily cigarettes while pregnant	−1.35	0.54	−2.42–(−)0.28	0.01	0.04
Mother’s age Wave 1	0.34	0.24	−0.12–0.81	0.15	0.37
Pregnancy: 2nd trimester days per week drank alcohol	5.24	4.28	−3.16–13.64	0.22	0.47
Pregnancy: Average number of drinks	−1.86	2.48	−6.73–3.00	0.45	0.75
Breastfeeding duration (days)	0.01	0.01	−0.01–0.02	0.45	0.75
Breastfeeding status (currently or previously breastfed)	−1.79	2.66	−7.04–3.45	0.50	0.75
Mother’s Average daily cigarettes Wave 1	−0.13	0.42	−0.97–0.70	0.76	0.98
Pregnancy: 1st trimester days per week drank alcohol	−0.29	2.74	−5.68–5.10	0.92	0.98
Pregnancy: 3rd trimester days per week drank alcohol	−0.23	3.70	−7.51–7.04	0.95	0.98
Child’s birth weight (grams)	<0.0001	<0.0001	<0.0001–<0.0001	0.97	0.98
Child’s age (months)	0.01	0.28	−0.54–0.56	0.98	0.98
Intercept	446.23	38.39	370.80–521.67	<0.0001	N/A

* Higher scores indicate lower income.

**Table 6:** Grade 3 NAPLAN grammar and punctuation scores (babies who had been breastfed at any time).

Variable	B	SE	95% CI	*P* Value	Benjamini-Hochberg *P* Value
Child’s sex	26.44	2.86	20.83–32.05	<0.0001	<0.0001
Combined family income *	−4.09	0.68	−5.42–(−)2.76	<0.0001	<0.0001
Mother’s level of education	11.22	1.12	9.03–13.42	<0.0001	<0.0001
Child’s age (months)	1.78	0.33	1.14–2.42	<0.0001	<0.0001
Mother’s age Wave 1	1.01	0.31	0.41–1.61	<0.0001	<0.0001
Mother’s modified AUDIT-C score Wave 1	−2.11	0.75	−3.59–(−)0.64	0.01	0.01
Average daily cigarettes while pregnant	−1.57	0.61	−2.76–(−)0.37	0.01	0.02
Breastfeeding status (currently or previously breastfed)	−4.50	3.20	−10.79–1.80	0.16	0.30
Child’s birth weight (grams)	<0.0001	<0.0001	<0.0001–0.01	0.28	0.47
Pregnancy: Average number of drinks	2.73	3.35	−3.86–9.33	0.42	0.62
Pregnancy: 3rd trimester days per week drank alcohol	2.93	4.40	−5.71–11.58	0.51	0.69
Mother’s Average daily cigarettes Wave 1	−0.28	0.46	−1.18–0.63	0.55	0.69
Breastfeeding duration (days)	<0.0001	0.01	−0.01–0.02	0.67	0.78
Pregnancy: 1st trimester days per week drank alcohol	0.30	3.46	−6.50–7.10	0.93	0.94
Pregnancy: 2nd trimester days per week drank alcohol	−0.42	5.39	−11.01–10.17	0.94	0.94
Intercept	142.44	38.84	66.17–218.70	<0.0001	N/A

* Higher scores indicate lower income.

## Supplementary Materials

The following are available online at https://www.mdpi.com/2072-6643/12/3/829/s1.
