# Supplementary material for: Drinking or Smoking While Breastfeeding and Later Academic Outcomes in Children
Source: Nutrients. 2020 Mar 20;12(3):829. doi: 10.3390/nu12030829 (PMC7146206; doi:10.3390/nu12030829)
Supplement: Supplementary file 1 [file nutrients-12-00829-s001.pdf]

S1: Wave 1 maternal alcohol consumption and tobacco smoking prior to MI.

Wave 1 modified AUDIT-C scores (Kolmogorov–Smirnov=0·132, df=3714, p<0·001) and the number of cigarettes smoked per day were skewed (Kolmogorov–Smirnov=0·48, df=4276, p<0·001). Maternal modified AUDIT-C scores were lower in babies who were actively breastfeeding at Wave 1 (Median=5, range=1-17, n=1490) than babies who had been previously breastfed (Median=6, range=1-15 n=1951; Mann-Whitney U=1253418·50, p=<0·0001). Similarly, mothers actively breastfeeding smoked fewer cigarettes (Median=0, range=0-30, n=1757) than mothers who had already stopped breastfeeding their babies (Median=0, range=0-40, n=2202; Mann-Whitney U= 1677797·50, p=<0·0001).

S2: NAPLAN reading scores.

Babies who were breastfeeding at Wave 1

Grade 3

Variable	B	SE	95% CI	P value	Benjamini-Hochberg P value
Combined family income*	-3·93	0·99	-5·88-(-)1·98	<0·0001	<0·0001
Mother's level of education	12·59	1·62	9·42-15·76	<0·0001	<0·0001
Child's age (months)	2·44	0·50	1·47-3·41	<0·0001	<0·0001
Child's sex	12·96	4·51	4·10-21·82	<0·0001	0·01
Mother's age Wave 1	1·01	0·45	0·12-1·90	0·03	0·07
Child's birth weight (grams)	0·01	<0·0001	<0·0001-0·02	0·07	0·17
Pregnancy: 3rd trimester days per week drank alcohol	-4·13	5·71	-15·33-7·08	0·47	0·73
Breastfeeding duration (days)	-0·01	0·01	-0·03-0·01	0·53	0·73
Average daily cigarettes while pregnant	-0·88	1·39	-3·61-1·85	0·53	0·73
Pregnancy: 1st trimester days per week drank alcohol	2·56	4·81	-6·87-12·00	0·59	0·73
Mother's modified AUDIT-C score Wave 1	0·52	1·11	-1·67-2·71	0·64	0·73
Pregnancy: 2nd trimester days per week drank alcohol	3·07	7·16	-10·97-17·11	0·67	0·73
Pregnancy: Average number of drinks	-2·10	5·12	-12·17-7·97	0·68	0·73
Mother's Average daily cigarettes Wave 1	-0·22	1·03	-2·25-1·81	0·83	0·83
Intercept	59·35	57·02	-52·51-171·22	0·30	N/A

*Higher scores indicate lower income

Grade 5

Variable	B	SE	95% CI	P value	Benjamini-Hochberg P value
Combined family income*	-3·77	0·90	-5·55-(-)2·00	<0·0001	<0·0001
Mother's level of education	12·41	1·45	9·55-15·27	<0·0001	<0·0001
Child's age (months)	1·05	0·48	0·12-1·99	0·03	0·13
Mother's age Wave 1	0·77	0·39	<0·0001-1·54	0·05	0·17
Child's sex	6·42	3·89	-1·21-14·04	0·10	0·28

Child's birth weight (grams)	0·01	<0·0001	<0·0001-0·01	0·14	0·33
Mother's modified AUDIT-C score Wave 1	1·04	0·99	-0·90-2·98	0·29	0·58
Mother's Average daily cigarettes Wave 1	-0·70	0·87	-2·42-1·02	0·43	0·68
Pregnancy: 1st trimester days per week drank alcohol	2·96	4·22	-5·33-11·25	0·48	0·68
Pregnancy: Average number of drinks	-2·48	4·43	-11·18-6·22	0·58	0·68
Breastfeeding duration (days)	-0·01	0·01	-0·03-0·01	0·58	0·68
Pregnancy: 3rd trimester days per week drank alcohol	-2·86	5·25	-13·16-7·44	0·59	0·68
Pregnancy: 2nd trimester days per week drank alcohol	0·96	6·53	-11·87-13·78	0·88	0·91
Average daily cigarettes while pregnant	-0·14	1·19	-2·46-2·19	0·91	0·91
Intercept	279·30	64·46	152·70-405·90	<0·0001	N/A

*Higher scores indicate lower income

<u>Babies who had been breastfed at any time</u>

Grade 3

**Variable**	**B**	**SE**	**95% CI**	**P value**	**Benjamini-Hochberg P value**
Child's sex	14·54	2·99	8·66-20·43	<0·0001	<0·0001
Combined family income*	-3·94	0·68	-5·28-(-)2·60	<0·0001	<0·0001
Mother's level of education	11·17	1·01	9·19-13·14	<0·0001	<0·0001
Child's age (months)	2·66	0·32	2·03-3·29	<0·0001	<0·0001
Breastfeeding status (currently or previously breastfed)	-7·95	3·02	-13·89-(-)2·01	0·01	0·03
Mother's age Wave 1	0·76	0·29	0·18-1·33	0·01	0·03
Child's birth weight (grams)	0·01	<0·0001	<0·0001-0·01	0·05	0·11
Average daily cigarettes while pregnant	-1·07	0·67	-2·38-0·24	0·11	0·21
Mother's Average daily cigarettes Wave 1	-0·46	0·47	-1·39-0·47	0·33	0·55
Mother's modified AUDIT-C score Wave 1	-0·25	0·71	-1·64-1·15	0·73	0·93
Pregnancy: Average number of drinks	1·07	3·34	-5·51-7·64	0·75	0·93
Pregnancy: 1st trimester days per week drank alcohol	0·78	3·43	-5·97-7·52	0·82	0·93
Pregnancy: 2nd trimester days per week drank alcohol	0·75	5·33	-9·74-11·24	0·89	0·93

Breastfeeding duration (days)	<0·0001	0·01	-0·02-0·01	0·90	0·93
Pregnancy: 3rd trimester days per week drank alcohol	-0·38	4·22	-8·67-7·92	0·93	0·93
Intercept	61·36	37·00	-11·28-133·99	0·10	N/A

*Higher scores indicate lower income

Grade 5

**Variable**	**B**	**SE**	**95% CI**	**P value**	**Benjamini-Hochberg P value**
Child's sex	9·16	2·58	4·09-14·23	<0·0001	<0·0001
Combined family income*	-3·57	0·55	-4·66-(-)2·49	<0·0001	<0·0001
Mother's level of education	10·82	0·92	9·02-12·63	<0·0001	<0·0001
Child's age (months)	0·99	0·31	0·38-1·59	<0·0001	<0·0001
Mother's age Wave 1	0·77	0·26	0·27-1·28	<0·0001	0·01
Breastfeeding status (currently or previously breastfed)	-8·14	2·76	-13·57-(-)2·72	<0·0001	0·01
Child's birth weight (grams)	0·01	<0·0001	<0·0001-0·01	0·04	0·08
Average daily cigarettes while pregnant	-1·16	0·57	-2·29-(-)0·03	0·05	0·08
Mother's Average daily cigarettes Wave 1	-0·27	0·42	-1·09-0·56	0·52	0·87
Pregnancy: 3rd trimester days per week drank alcohol	1·90	3·92	-5·82-9·62	0·63	0·92
Pregnancy: 2nd trimester days per week drank alcohol	-1·36	4·69	-10·58-7·87	0·77	0·92
Breastfeeding duration (days)	<0·0001	0·01	-0·01-0·01	0·82	0·92
Pregnancy: Average number of drinks	-0·56	2·74	-5·95-4·83	0·84	0·92
Mother's modified AUDIT-C score Wave 1	-0·11	0·61	-1·30-1·08	0·86	0·92
Pregnancy: 1st trimester days per week drank alcohol	0·30	2·82	-5·25-5·84	0·92	0·92
Intercept	298·19	42·55	214·49-381·88	<0·0001	N/A

*Higher scores indicate lower income

S3: NAPLAN writing scores.

<u>Babies who were breastfeeding at Wave 1</u>

Grade 3

**Variable**	**B**	**SE**	**95% CI**	**P value**	**Benjamini-Hochberg P value**
Child's sex	26·28	3·15	20·10-32·47	<0·0001	<0·0001
Combined family income*	-3·31	0·66	-4·60-(-)2·02	<0·0001	<0·0001
Mother's level of education	6·61	1·07	4·50-8·72	<0·0001	<0·0001
Child's age (months)	1·32	0·35	0·63-2·01	<0·0001	<0·0001
Mother's age Wave 1	0·49	0·30	-0·11-1·08	0·11	0·31
Average daily cigarettes while pregnant	-1·15	1·00	-3·12-0·82	0·25	0·53
Mother's modified AUDIT-C score Wave 1	-0·84	0·75	-2·31-0·63	0·26	0·53
Child's birth weight (grams)	<0·0001	<0·0001	<0·0001-0·01	0·39	0·67
Breastfeeding duration (days)	0·01	0·01	-0·01-0·02	0·45	0·67
Pregnancy: 2nd trimester days per week drank alcohol	3·36	4·78	-6·01-12·72	0·48	0·67
Pregnancy: 3rd trimester days per week drank alcohol	-1·97	3·84	-9·50-5·55	0·61	0·77
Mother's average. daily cigarettes Wave 1	-0·23	0·72	-1·66-1·20	0·75	0·87
Pregnancy: Average. number of drinks	-0·02	3·44	-6·78-6·74	>0·99	>0·99
Pregnancy: 1st trimester days per week drank alcohol	0·01	3·16	-6·18-6·20	>0·99	>0·99
Intercept	208·79	39·44	131·33-286·25	<0·0001	N/A

*Higher scores indicate lower income

Grade 5

**Variable**	**B**	**SE**	**95% CI**	**P value**	**Benjamini-Hochberg P value**
Child's sex	27·89	3·37	21·26-34·51	<0·0001	<0·0001
Combined family income*	-3·16	0·70	-4·53-(-)1·78	<0·0001	<0·0001
Mother's level of education	6·62	1·27	4·12-9·13	<0·0001	<0·0001
Mother's age Wave 1	0·77	0·34	0·11-1·43	0·02	0·08
Pregnancy: 2nd trimester days per week drank alcohol	8·37	5·46	-2·35-19·10	0·13	0·32

Child's age (months)	0·59	0·41	-0·22-1·41	0·15	0·32
Pregnancy: Average number of drinks	-5·11	3·68	-12·34-2·13	0·17	0·32
Mother's Average daily cigarettes Wave 1	-1·00	0·74	-2·46-0·46	0·18	0·32
Pregnancy: 1st trimester days per week drank alcohol	-3·69	3·44	-10·44-3·07	0·29	0·44
Breastfeeding duration (days)	<0·0001	0·01	-0·02-0·01	0·68	0·86
Pregnancy: 3rd trimester days per week drank alcohol	-1·71	4·29	-10·13-6·71	0·69	0·86
Mother's modified AUDIT-C score Wave 1	-0·23	0·84	-1·87-1·41	0·78	0·86
Average daily cigarettes while pregnant	-0·27	1·05	-2·34-1·79	0·79	0·86
Child's birth weight (grams)	<0·0001	<0·0001	-0·01-0·01	>0·99	>0·99
Intercept	319·59	53·96	213·50-425·67	<0·0001	N/A

*Higher scores indicate lower income

<u>Babies who had breastfed at any time</u>

Grade 5

**Variable**	**B**	**SE**	**95% CI**	**P value**	**Benjamini-Hochberg P value**
Child's sex	28·63	2·13	24·44-32·82	<0·0001	<0·0001
Combined family income*	-3·32	0·48	-4·26-(-)2·37	<0·0001	<0·0001
Mother's level of education	6·30	0·78	4·77-7·83	<0·0001	<0·0001
Mother's age Wave 1	0·70	0·24	0·23-1·18	<0·0001	0·02
Pregnancy: Average number of drinks	-6·80	2·39	-11·50-(-)2·09	0·01	0·02
Mother's Average daily cigarettes Wave 1	-0·67	0·39	-1·44-0·11	0·09	0·21
Mother's modified AUDIT-C score Wave 1	-0·85	0·51	-1·85-0·16	0·10	0·21
Pregnancy: 2nd trimester days per week drank alcohol	5·80	4·05	-2·17-13·78	0·15	0·29
Pregnancy: 1st trimester days per week drank alcohol	-3·18	2·49	-8·07-1·71	0·20	0·31
Child's age (months)	0·35	0·28	-0·19-0·90	0·21	0·31
Average daily cigarettes while pregnant	-0·69	0·57	-1·81-0·44	0·23	0·31
Child's birth weight (grams)	<0·0001	<0·0001	<0·0001-0·01	0·28	0·35
Breastfeeding duration (days)	-0·01	0·01	-0·02-0·01	0·34	0·40

Breastfeeding status (currently or previously breastfed)	-1·29	2·16	-5·52-2·95	0·55	0·59
Pregnancy: 3rd trimester days per week drank alcohol	0·89	3·36	-5·73-7·52	0·79	0·79
Intercept	350·46	37·86	275·80-425·11	<0·0001	N/A

*Higher scores indicate lower income

S4: NAPLAN spelling scores.

Babies who were breastfeeding at Wave 1

Grade 3

Variable	B	SE	95% CI	P value	Benjamini-Hochberg P value
Child's sex	23·09	3·91	15·42-30·76	<0·0001	<0·0001
Combined family income*	-3·09	0·84	-4·74-(-)1·44	<0·0001	<0·0001
Mother's level of education	8·03	1·36	5·36-10·71	<0·0001	<0·0001
Child's age (months)	1·52	0·44	0·66-2·38	<0·0001	<0·0001
Average daily cigarettes while pregnant	-2·21	1·18	-4·53-0·11	0·06	0·17
Mother's modified AUDIT-C score Wave 1	-1·60	0·96	-3·50-0·29	0·10	0·21
Mother's age Wave 1	0·66	0·40	-0·13-1·45	0·10	0·21
Mother's Average daily cigarettes Wave 1	0·87	0·87	-0·83-2·58	0·32	0·55
Pregnancy: 1st trimester days per week drank alcohol	2·28	4·07	-5·70-10·26	0·58	0·79
Pregnancy: Average number of drinks	-2·13	4·33	-10·64-6·38	0·62	0·79
Pregnancy: 3rd trimester days per week drank alcohol	1·58	4·97	-8·17-11·32	0·75	0·79
Pregnancy: 2nd trimester days per week drank alcohol	1·94	6·14	-10·09-13·97	0·75	0·79
Child's birth weight (grams)	<0·0001	<0·0001	-0·01-0·01	0·75	0·79
Breastfeeding duration (days)	<0·0001	0·01	-0·02-0·02	0·79	0·79
Intercept	180·58	50·44	81·58-279·58	<0·0001	N/A

*Higher scores indicate lower income

Grade 5

Variable	B	SE	95% CI	P value	Benjamini-Hochberg P value
Child's sex	16·77	3·73	9·43-24·11	<0·0001	<0·0001
Combined family income*	-2·99	0·76	-4·47-(-)1·51	<0·0001	<0·0001
Mother's level of education	7·89	1·35	5·24-10·55	<0·0001	<0·0001
Mother's modified AUDIT-C score Wave 1	-1·11	0·90	-2·88-0·66	0·22	0·69

Average daily cigarettes while pregnant	-1·23	1·14	-3·48-1·02	0·28	0·69
Pregnancy: 2nd trimester days per week drank alcohol	6·36	6·05	-5·52-18·25	0·29	0·69
Mother's age Wave 1	0·34	0·37	-0·39-1·06	0·37	0·73
Breastfeeding duration (days)	<0·0001	0·01	-0·01-0·02	0·67	0·90
Pregnancy: 3rd trimester days per week drank alcohol	-2·03	4·84	-11·54-7·49	0·68	0·90
Pregnancy: Average number of drinks	-1·29	3·88	-8·90-6·33	0·74	0·90
Child's age (months)	0·08	0·44	-0·78-0·95	0·85	0·90
Child's birth weight (grams)	<0·0001	<0·0001	-0·01-0·01	0·86	0·90
Pregnancy: 1st trimester days per week drank alcohol	0·67	3·84	-6·87-8·20	0·86	0·90
Mother's Average daily cigarettes Wave 1	0·10	0·84	-1·55-1·75	0·90	0·90
Intercept	428·49	58·60	313·41-543·57	<0·0001	N/A

*Higher scores indicate lower income

S5: NAPLAN grammar and punctuation scores.

Babies who were breastfeeding at Wave 1

Grade 3

Variable	B	SE	95% CI	P value	Benjamini-Hochberg P value
Child's sex	26·22	4·28	17·82-34·62	<0·0001	<0·0001
Combined family income*	-4·05	0·99	-6·00-(-)2·11	<0·0001	<0·0001
Mother's level of education	12·44	1·71	9·08-15·81	<0·0001	<0·0001
Child's age (months)	1·44	0·50	0·46-2·42	<0·0001	0·01
Mother's age Wave 1	0·74	0·45	-0·15-1·63	0·10	0·29
Average daily cigarettes while pregnant	-2·07	1·37	-4·76-0·62	0·13	0·30
Mother's modified AUDIT-C score Wave 1	-1·59	1·10	-3·75-0·57	0·15	0·30
Child's birth weight (grams)	0·01	<0·0001	<0·0001-0·01	0·19	0·33
Pregnancy: Average number of drinks	4·52	4·99	-5·28-14·33	0·37	0·57
Pregnancy: 1st trimester days per week drank alcohol	2·41	4·76	-6·92-11·75	0·61	0·86
Pregnancy: 3rd trimester days per week drank alcohol	1·59	5·74	-9·67-12·85	0·78	0·93
Mother's Average daily cigarettes Wave 1	0·26	1·01	-1·74-2·25	0·80	0·93
Breastfeeding duration (days)	<0·0001	0·01	-0·02-0·02	0·89	0·96
Pregnancy: 2nd trimester days per week drank alcohol	0·15	7·14	-13·85-14·15	0·98	0·98
Intercept	166·82	56·22	56·54-277·10	<0·0001	N/A

*Higher scores indicate lower income

Grade 5

Variable	B	SE	95% CI	P value	Benjamini-Hochberg P value
Child's sex	17·73	4·30	9·28-26·19	<0·0001	<0·0001
Mother's level of education	13·92	1·61	10·75-17·09	<0·0001	<0·0001
Combined family income*	-3·24	0·94	-5·09-(-)1·39	<0·0001	<0·0001
Pregnancy: 2nd trimester days per week drank alcohol	11·52	6·80	-1·83-24·87	0·09	0·32

Pregnancy: 3rd trimester days per week drank alcohol	-8·12	5·35	-18·63-2·39	0·13	0·36
Child's birth weight (grams)	0·01	<0·0001	<0·0001-0·01	0·17	0·38
Mother's age Wave 1	0·56	0·42	-0·27-1·39	0·19	0·38
Child's age (months)	0·52	0·50	-0·47-1·51	0·30	0·53
Mother's Average daily cigarettes Wave 1	-0·53	0·91	-2·32-1·27	0·57	0·86
Average daily cigarettes while pregnant	-0·63	1·28	-3·15-1·89	0·62	0·86
Mother's modified AUDIT-C score Wave 1	0·32	1·05	-1·75-2·39	0·76	0·86
Breastfeeding duration (days)	<0·0001	0·01	-0·02-0·02	0·80	0·86
Pregnancy: 1st trimester days per week drank alcohol	-0·81	4·39	-9·44-7·81	0·85	0·86
Pregnancy: Average number of drinks	-0·84	4·59	-9·86-8·19	0·86	0·86
Intercept	325·77	67·44	193·27-458·28	<0·0001	N/A

*Higher scores indicate lower income

<u>Babies who had been breastfed at any time</u>

Grade 5

**Variable**	**B**	**SE**	**95% CI**	**P value**	**Benjamini-Hochberg P value**
Child's sex	15·74	2·78	10·27-21·20	<0·0001	<0·0001
Combined family income*	-3·42	0·62	-4·64-(-)2·20	<0·0001	<0·0001
Mother's level of education	12·01	1·00	10·04-13·98	<0·0001	<0·0001
Mother's age Wave 1	0·79	0·26	0·28-1·29	<0·0001	0·01
Average daily cigarettes while pregnant	-1·59	0·62	-2·81-(-)0·36	0·01	0·03
Breastfeeding status (currently or previously breastfed)	-7·00	2·95	-12·79-(-)1·21	0·02	0·05
Pregnancy: 2nd trimester days per week drank alcohol	8·00	4·89	-1·60-17·61	0·10	0·22
Pregnancy: 3rd trimester days per week drank alcohol	-4·49	4·00	-12·34-3·36	0·26	0·46
Mother's modified AUDIT-C score Wave 1	-0·67	0·65	-1·94-0·61	0·30	0·46
Child's birth weight (grams)	<0·0001	<0·0001	<0·0001-0·01	0·31	0·46
Child's age (months)	0·29	0·33	-0·36-0·95	0·38	0·52
Mother's Average daily cigarettes Wave 1	-0·32	0·44	-1·19-0·55	0·47	0·59

Breastfeeding duration (days)	<0·0001	0·01	-0·01-0·02	0·65	0·74
Pregnancy: 1st trimester days per week drank alcohol	-0·95	3·18	-7·20-5·30	0·77	0·82
Pregnancy: Average number of drinks	-0·50	2·96	-6·31-5·31	0·87	0·87
Intercept	377·34	45·16	288·50-466·18	<0·0001	N/A

*Higher scores indicate lower income

S6: NAPLAN numeracy scores.

Babies who were breastfeeding at Wave 1

Grade 3

Variable	B	SE	95% CI	P value	Benjamini-Hochberg P value
Combined family income*	-3·39	0·77	-4·91-(-)1·86	<0·0001	<0·0001
Mother's level of education	9·56	1·27	7·06-12·07	<0·0001	<0·0001
Child's age (months)	1·92	0·41	1·11-2·72	<0·0001	<0·0001
Mother's age Wave 1	1·04	0·37	0·32-1·77	0·01	0·02
Child's birth weight (grams)	0·01	<0·0001	<0·0001-0·01	0·02	0·06
Child's sex	-7·12	3·48	-13·95-(-)0·30	0·04	0·10
Average daily cigarettes while pregnant	-1·78	1·15	-4·05-0·49	0·12	0·25
Pregnancy: Average number of drinks	4·90	4·20	-3·38-13·17	0·25	0·43
Pregnancy: 1st trimester days per week drank alcohol	3·49	3·63	-3·63-10·60	0·34	0·52
Mother's modified AUDIT-C score Wave 1	-0·74	0·90	-2·51-1·04	0·41	0·58
Pregnancy: 3rd trimester days per week drank alcohol	-1·91	4·45	-10·63-6·81	0·67	0·85
Breastfeeding duration (days)	<0·0001	0·01	-0·02-0·02	0·76	0·88
Mother's Average daily cigarettes Wave 1	0·19	0·81	-1·42-1·79	0·82	0·88
Pregnancy: 2nd trimester days per week drank alcohol	-0·41	5·78	-11·75-10·94	0·94	0·94
Intercept	131·16	46·30	40·27-222·05	0·01	N/A

*Higher scores indicate lower income

Grade 5

Variable	B	SE	95% CI	P value	Benjamini-Hochberg P value
Child's sex	-13·09	3·44	-19·85-(-)6·33	<0·0001	<0·0001
Combined family income*	-3·18	0·81	-4·78-(-)1·59	<0·0001	<0·0001
Mother's level of education	10·42	1·27	7·92-12·93	<0·0001	<0·0001
Mother's age Wave 1	0·77	0·35	0·08-1·45	0·03	0·10
Child's birth weight (grams)	0·01	<0·0001	<0·0001-0·01	0·07	0·18

Pregnancy: 2nd trimester days per week drank alcohol	10·20	5·87	-1·33-21·73	0·08	0·19
Pregnancy: 3rd trimester days per week drank alcohol	-6·62	4·38	-15·21-1·97	0·13	0·26
Child's age (months)	0·60	0·44	-0·27-1·47	0·17	0·30
Average daily cigarettes while pregnant	-1·16	1·10	-3·31-1·00	0·29	0·44
Pregnancy: Average number of drinks	3·91	3·88	-3·71-11·53	0·31	0·44
Mother's modified AUDIT-C score Wave 1	-0·65	0·88	-2·38-1·07	0·46	0·58
Pregnancy: 1st trimester days per week drank alcohol	-1·02	3·79	-8·46-6·42	0·79	0·90
Mother's Average daily cigarettes Wave 1	0·16	0·78	-1·37-1·69	0·84	0·90
Breastfeeding duration (days)	<0·0001	0·01	-0·02-0·02	0·90	0·90
Intercept	357·68	58·44	242·79-472·57	<0·0001	N/A

*Higher scores indicate lower income

<u>Babies who had been breastfed at any time</u>

Grade 3

**Variable**	**B**	**SE**	**95% CI**	**P value**	**Benjamini-Hochberg P value**
Combined family income*	-3·34	0·51	-4·35-(-)2·33	<0·0001	<0·0001
Mother's level of education	8·92	0·85	7·26-10·59	<0·0001	<0·0001
Child's age (months)	1·76	0·27	1·24-2·28	<0·0001	<0·0001
Child's birth weight (grams)	0·01	<0·0001	<0·0001-0·01	<0·0001	<0·0001
Mother's age Wave 1	0·82	0·24	0·35-1·29	<0·0001	<0·0001
Child's sex	-7·04	2·36	-11·68-(-)2·40	<0·0001	0·01
Average daily cigarettes while pregnant	-1·30	0·60	-2·48-(-)0·11	0·03	0·07
Pregnancy: Average number of drinks	5·31	2·64	0·13-10·50	0·04	0·08
Mother's modified AUDIT-C score Wave 1	-1·13	0·58	-2·28-0·02	0·05	0·09
Breastfeeding status (currently or previously breastfed)	-2·17	2·41	-6·89-2·55	0·37	0·55
Mother's Average daily cigarettes Wave 1	-0·31	0·42	-1·13-0·51	0·45	0·62
Pregnancy: 2nd trimester days per week drank alcohol	-1·51	4·36	-10·07-7·05	0·73	0·90

Pregnancy: 3rd trimester days per week drank alcohol	1·02	3·61	-6·08-8·12	0·78	0·90
Pregnancy: 1st trimester days per week drank alcohol	0·37	2·66	-4·86-5·59	0·89	0·91
Breastfeeding duration (days)	<0·0001	0·01	-0·01-0·01	0·91	0·91
Intercept	161·30	31·13	100·14-222·46	<0·0001	N/A

*Higher scores indicate lower income

Grade 5

**Variable**	**B**	**SE**	**95% CI**	**P value**	**Benjamini-Hochberg P value**
Child's sex	-13·49	2·30	-18·00-(-)8·97	<0·0001	<0·0001
Combined family income*	-3·13	0·51	-4·14-(-)2·12	<0·0001	<0·0001
Mother's level of education	9·81	0·83	8·17-11·44	<0·0001	<0·0001
Child's birth weight (grams)	0·01	<0·0001	<0·0001-0·01	<0·0001	<0·0001
Mother's age Wave 1	0·73	0·23	0·29-1·18	<0·0001	<0·0001
Mother's modified AUDIT-C score Wave 1	-1·24	0·55	-2·32-(-)0·17	0·02	0·06
Average daily cigarettes while pregnant	-1·18	0·61	-2·39-0·04	0·06	0·12
Child's age (months)	0·51	0·28	-0·04-1·06	0·07	0·13
Pregnancy: 2nd trimester days per week drank alcohol	6·03	4·21	-2·24-14·31	0·15	0·25
Pregnancy: Average number of drinks	3·09	2·59	-2·02-8·19	0·24	0·35
Pregnancy: 1st trimester days per week drank alcohol	-2·80	2·55	-7·82-2·21	0·27	0·37
Breastfeeding status (currently or previously breastfed)	-2·18	2·42	-6·92-2·57	0·37	0·46
Pregnancy: 3rd trimester days per week drank alcohol	-1·97	3·48	-8·81-4·86	0·57	0·66
Breastfeeding duration (days)	<0·0001	0·01	-0·01-0·01	0·78	0·84
Mother's Average daily cigarettes Wave 1	-0·01	0·40	-0·80-0·77	0·97	0·97
Intercept	374·00	38·83	297·57-450·43	<0·0001	N/A

*Higher scores indicate lower income
